# Influence of Solvent, Electron Acceptors and Arenes on Photochemical Decarboxylation of Free Carboxylic Acids via Single Electron Transfer (SET)

**DOI:** 10.3390/molecules15042623

**Published:** 2010-04-12

**Authors:** Yasuharu Yoshimi, Shota Hayashi, Keisuke Nishikawa, Yoshiki Haga, Kousuke Maeda, Toshio Morita, Tatsuya Itou, Yutaka Okada, Nobuyuki Ichinose, Minoru Hatanaka

**Affiliations:** 1 Department of Applied Chemistry and Biotechnology, Graduate School of Engineering, University of Fukui, 3-9-1 Bunkyo, Fukui 910-8507, Japan; 2 Department of Applied Chemistry, College of Life Sciences, Ritsumeikan University, 1-1-1 Noji-Higashi, Kusatsu 525-8577, Japan; E-Mail: t-itou@fc.ritsumei.ac.jp (T.I.); 3 Department of Chemistry and Materials Technology, Kyoto Institue of Technology, Matsugasaki, Sakyo-ku, Kyoto 606-8585, Japan; E-Mail: ichinose@kit.ac.jp (N.I.); 4 Department of Medicinal and Organic Chemistry, School of Pharmacy, Iwate Medical University, Yahaba, Iwate 028-3694, Japan; E-Mail: mhatanak@iwate-med.ac.jp (M.H.)

**Keywords:** photochemical decarboxylation, single electron transfer, free carboxylic acid

## Abstract

Single electron transfer (SET)-photochemical decarboxylation of free carboxylic acids was performed in a polar solvent using several arenes such as phenanthrene, naphthalene, 1-methylnaphthalene, biphenyl, triphenylene, and chrysene in the presence of various electron acceptors such as 1,2-, 1,3-, and 1,4-dicyanobenzenes, methyl 4-cyanobenzoate, and 1,4-dicyanonaphthalene. The decarboxylation reaction was influenced by the arenes, electron acceptors, and solvent. The best result was achieved by the photoreaction using biphenyl and 1,4-dicyanonaphthalene in aqueous acetonitrile.

## 1. Introduction 

Decarboxylation of carboxylic acids (RCO_2_H) is one of the most important functional group transformations in organic synthesis, because carboxylic acids are found widely in Nature and are also commercially available. This transformation is usually performed by radical methods such as the Kolbe electrolysis and the Barton decarboxylation. The Kolbe electrolysis involves the anodic oxidation of a carboxylate ion (RCO_2_**^–^**) in which alkyl radicals are formed via the decarboxylation of carboxy radicals [1]. This reaction has been successfully utilized for C-C bond formation; however, it is limited in scope to producing the dimer (R-R) or the solvent adduct (ROH or ROR’) via further oxidation. The most efficient method is the Barton decarboxylation that involves the formation of alkyl radicals by homolysis of thiohydroxamic esters under either thermal or photochemical conditions [2,3]; however, this requires the esterification of carboxylic acids to thiohydroxamic esters as precursors. In addition to these methods, free carboxylic acids are also known to undergo decarboxylation via photoinduced electron transfer (PET) in the presence of various electron acceptors such as iminium salts [4], acridine [5], phthalimide [6,7], 1-cyanonaphthalene [8], and tetracyanobenzene [9] or heterogeneous photocatalysts such as TiO_2_ [10]. However, the carboxylic acids used in these cases have been limited to aryl, vinyl, and aryloxy acetic acids, which are relatively easy to oxidize. Thus, the generation of alkyl radicals from free carboxylic acids under mild conditions is still desired.

**Scheme 1:**
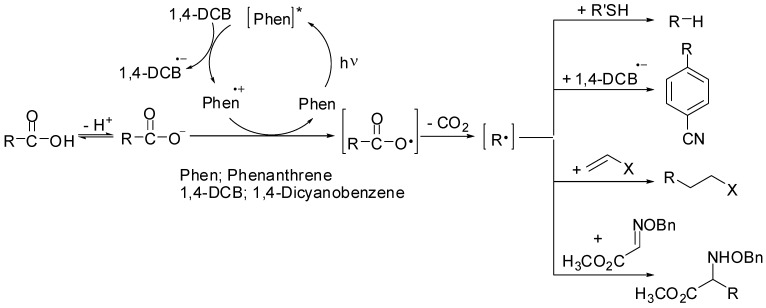
Decarboxylation of free carboxylic acid with the photogenerated cation radical of phenanthrene.

We have recently reported that the decarboxylation of free carboxylic acids by the photogenerated cation radical of phenanthrene (Phen) in a redox-photosensitized reaction system [11,12,13] produced alkyl radicals (Scheme 1) [14,15,16,17]. This process is promoted by a single electron transfer (SET) from the carboxylate ion to the cation radical of Phen formed by PET with 1,4-dicyanobenzene (1,4-DCB). This leads to the formation of the carboxy radical, which is then decarboxylated to produce an alkyl radical. The generated alkyl radicals react with the thiol [14], the anion radical of 1,4-DCB [15], alkenes [16], and oxime ether [17] to yield the corresponding reduction, substitution, and addition products, respectively. Thus, this photoreaction was proven to provide an efficient method for generating alkyl radicals from free aliphatic carboxylic acids under mild conditions. In this study, we endeavored to investigate the effects of solvent, electron acceptor, and arene on the photochemical decarboxylation.

## 2. Results and Discussion 

### 2.1. Solvent Effect

Initially, photodecarboxylative addition of *N*-Boc-l-valine **1** (Boc = *tert*-butoxycarbonyl) to acrylonitrile (**2**) in a variety of solvents was examined, as shown in Table 1. Excitation under an argon atmosphere of aqueous acetonitrile solutions (CH_3_CN/H_2_O = 9:1) containing Phen (20 mM), 1,4-DCB (20 mM), **1** (20 mM), and **2** (20 mM) with a 100-W high-pressure mercury lamp through a Pyrex filter (>300 nm) for 6 h at room temperature afforded **3** as a racemic mixture in 85% yield, along with recovery of Phen and 1,4-DCB (>90%) (entry 1 in Table 1). Catalytic amounts of Phen and 1,4-DCB (5 or 1 mM) under the same conditions required a long irradiation time (12 or 15 h) to produce a good yield of **3** (entries 2 and 3). The use of dry acetonitrile, DMF, methanol, and DMSO in place of aqueous acetonitrile as a solvent decreased the yield of **3** (entries 4**–**7), and less polar solvents such as 1,4-dioxane, THF, and benzene failed to produce the adduct **3**, with near-quantitative recovery of **1** (entries 8**–**10). Thus, the photochemical decarboxylation required a polar medium for forming the cation radical of Phen, and a higher yield of **3** was observed in aqueous acetonitrile.

### 2.2. Dependence on Electron Acceptor and Arene

Next, the photoreaction of **1** with **2** was examined using various electron acceptors and arenes (Table 2). Photodecarboxylation with 1,2-dicyanobenzene, 1,3-dicyanobenzene, methyl 4-cyano-benzoate, or 1,4-dicyanonaphthalene (1,4-DCN) as an electron acceptor in the presence of Phen occurred readily in aqueous acetonitrile (entries 1**–**4). In the case of methyl 4-cyanobenzoate, its low ability as an electron acceptor caused a decrease in the yield of **3** (entry 3). When a variety of arenes such as naphthalene, 1-methylnaphthalene, and biphenyl were used with 1,4-DCB, the adduct **3** was also obtained in a yield similar to that obtained with Phen and 1,4-DCB (entries 5**–**7); however, the use of pyrene (entry 8) did not produce **3**. This result indicated that a lower oxidation potential of the arene than that of the carboxylate ion resulted in no occurrence of SET between the cation radical of the arene and the carboxylate ion. In fact, the oxidation potential of an aliphatic carboxylate ion such as the hexanoate ion (+1.16 V *vs.* SCE in acetonitrile) [18] is similar to that of pyrene (+1.16 V *vs.* SCE in acetonitrile), yet the cation radical of pyrene did not oxidize the carboxylate ion in acetonitrile [19]. Thus, the decarboxylation was dependent on the oxidation potential of the arene. Similar photoreactions using arenes in the presence of 1,4-DCN also led to the formation of the adduct **3** in a yield similar to that obtained with Phen and 1,4-DCN (entries 9**–**11), except in the case of pyrene (entry 12). The use of 1,4-DCN as an electron acceptor led to the competitive absorption of arene and 1,4-DCN at 313 nm; however, the yield of **3** similar to that obtained with 1,4-DCB was observed. It is noteworthy that the yield of **3** was improved by using biphenyl and 1,4-DCN (entry 11).

The other arenes such as triphenylene and chrysene did not dissolve in aqueous acetonitrile, but dissolved in DMF, which led us to investigate the photoreaction using arenes with 1,4-DCB or 1,4-DCN in DMF. Table 3 shows that the photoreaction of **1** using triphenylene and chrysene in the presence of 1,4-DCB in DMF proceeded (entries 1 and 2), and, in the case of chrysene, a high yield of **3** was observed (entry 2). The use of naphthalene and 1-methylnaphthalene slightly decreased the yield of **3**, compared with that obtained in aqueous acetonitrile (entries 3 and 4). In particular, the use of biphenyl resulted in a low yield of **3** (entry 5). As reported earlier [21], the photoexcited 1,4-DCB reacted with DMF to yield a substituted product. The higher absorption of chrysene than 1,4-DCB at 313 nm prevented the photoreaction of 1,4-DCB with DMF to produce a higher yield of **3**; however, absorption of biphenyl did not occur at 313 nm, which produced photochemically excited 1,4-DCB, and this led to a low yield of **3**. Interestingly, the use of pyrene yielded the adduct **3** in the presence of 1,4-DCB or 1,4-DCN in DMF (entries 6 and 12), in contrast to the results obtained in aqueous acetonitrile. This indicates that the DMF-induced increase and decrease, respectively, of the oxidation potentials for pyrene and the carboxylate ion of **1** caused the SET between the cation radical of pyrene and the carboxylate ion of **1** to form the radical via decarboxylation. On the other hand, the use of 1,4- DCN in DMF significantly decreased the yield of **3** (entries 7**–**11), except in the case of chrysene. In addition, the recovery of 1,4-DCN was very low (10**–**30%). As with 1,4-DCB, these results could be attributed to the photoreaction of 1,4-DCN with DMF preventing decarboxylation, although the reaction of the photoexcited 1,4-DCN and DMF has not been reported. A detailed study of this photoreaction of 1,4-DCN with DMF is now in progress in our laboratory and will be reported in separate publications. Thus, decarboxylation was influenced by the arene, electron acceptor, and solvent.

## 3. Experimental Section

### 3.1. General

The melting point was measured on a hot stage apparatus and is uncorrected. The IR spectrum was recorded on a JASCO FT/IR-620 spectrometer. ^1^H- and ^13^C-NMR spectra were recorded on a JEOL JNM-AL500 (500 and 125 MHz) spectrometer for solutions in CDCl_3_ containing tetramethylsilane as an internal standard. The high resolution mass spectrum (HRMS) was obtained on a JEOL JMS-700T mass spectrometer. The light source was a Riko UV-100HA 100-W high-pressure mercury arc. Dicyanobenzenes and arenes were recrystallized from hexane and EtOAc.

### 3.2. General Procedure for the Photoreaction of N-Boc-l-Valine *(**1**)* to Acrylonitrile *(**2**):* Preparation of N-tert-butoxycarbonyl-4-amino-4-isopropylbutanenitrile *(**3**)*

An aqueous CH_3_CN solution (CH_3_CN 54 mL, H_2_O 6 mL) of **1** (1.2 mmol, 20 mM), Phen (1.2 mmol, 20 mM), and 1,4-DCB (1.2 mmol, 20 mM) in four Pyrex vessels (18 mm × 180 mm) was purged with argon for 10 min, and **2** (1.2 mmol, 20 mM) was added under argon atmosphere. The mixture was irradiated with a 100-W high-pressure mercury lamp for 6 h. The solvent was then evaporated, and the resulting residue was dissolved in ether and washed with water, dried over Na_2_SO_4_, and concentrated under reduced pressure to yield the adduct **3**. This product was isolated by column chromatography on a silica gel using hexane and EtOAc as eluents and by preparative HPLC using a GPC column. White solid; m.p. 76 °C; IR (KBr, cm^-1^) 3383, 2964, 2249, 1680, 1512; ^1^H-NMR: δ 4.34 (d (br), 0.75H), 4.03 (s (br), 0.10H), 3.48-3.43 (m, 1H), 2.46–2.34 (m, 2H), 1.91 (m, 1H), 1.71 (m, 1H), 1.62 (m, 1H), 1.44 (s, 9H), 0.94–0.90 (m, 6H); ^13^C-NMR: δ 155.9, 119.8, 79.6, 55.2, 32.3, 29.2, 28.3, 19.1, 17.8, 14.6; HRMS (FAB) calcd for (M+H)^+^ C_12_H_23_N_2_O_2_: 227.1761, found: 227.1738.

## 4. Conclusions 

In conclusion, we have found that several arenes and electron acceptors could be used in the SET-photochemical decarboxylation of free carboxylic acids. The photoreaction proceeded smoothly in a polar solvent and was highly dependent on the oxidation potential of the arenes used. The highest yield of **3** was achieved by using biphenyl and 1,4-DCN in aqueous acetonitrile. Further investigation of the application of this photoreaction is currently in progress.

## Figures and Tables

**Table 1:**
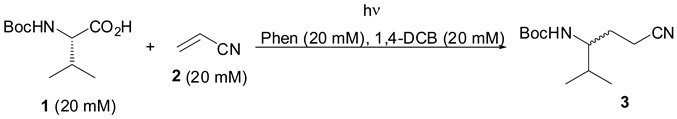
Solvent effect on photoreaction of **1** and **2** via decarboxylation.

Entry	Solvent	Yield of **3** / %^a^
1	CH_3_CN/H_2_O = 9:1	85
2^b^		77
3^c^		60
4	CH_3_CN	53
5	DMF	68
6	CH_3_OH	32
7	DMSO	13
8	1,4-Dioxane	0
9	THF	0
10	Benzene	0

^a^ Isolated yield. ^b^ Conditions: Phen (5 mM), 1,4-DCB (5 mM), 12 h.^c^Conditions: Phen (1 mM), 1,4-DCB (1 mM), 15 h.

**Table 2:**
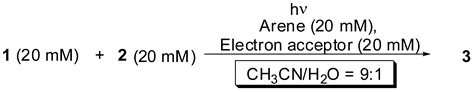
Photoreaction of **1** and **2** using several arenes and electron acceptors in aqueous acetonitrile.

Entry	Arene	Oxidation potential of arene / V^a^	Electron acceptor	Yield of **3** / %^b^
1	Phenanthrene	+ 1.50	1,2-DCB	73
2			1,3-DCB	80
3			Methyl 4-cyanobenzoate	64
4			1,4-DCN	82
5	Naphthalene	+ 1.70	1,4-DCB	77
6	1-Methylnaphthalene	+ 1.43		73
7	Biphenyl			87
8	Pyrene	+ 1.16		0
9	Naphthalene		1,4-DCN	75
10	1-Methylnaphthalene			80
11	Biphenyl			93
12	Pyrene			0

^a^Oxidation potentials *vs.* SCE in acetonitrile. Values compiled in Ref. [20]. ^b^Isolated yield.

**Table 3:**
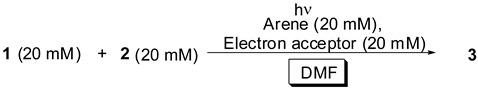
Photoreaction of **1** and **2** using several arenes and electron acceptors in DMF.

Entry	Arene	Oxidation potential of arene / V^a^	Electron acceptor	Yield of **3** / %^b^
1	Triphenylene	+ 1.55	1,4-DCB	44
2	Chrysene	+ 1.35		80
3	Naphthalene			71
4	1-Methylnaphthalene			68
5	Biphenyl			7
6	Pyrene			27
7	Triphenylene		1,4-DCN	19
8	Chrysene			74
9	Naphthalene			10
10	1-Methylnaphthalene			14
11	Biphenyl			6
12	Pyrene			22

^a^ Oxidation potentials *vs.* SCE in acetonitrile. Values compiled in Ref. [20]. ^b^Isolated yield.
